# GW4064 Alters Gut Microbiota Composition and Counteracts Autism-Associated Behaviors in BTBR T+tf/J Mice

**DOI:** 10.3389/fcimb.2022.911259

**Published:** 2022-06-22

**Authors:** Jiayin Liu, Chuanqi Liu, Zhanyuan Gao, Lianyu Zhou, Junwei Gao, Yi Luo, Tianyao Liu, Xiaotang Fan

**Affiliations:** ^1^ Department of Military Cognitive Psychology, School of Psychology, Third Military Medical University (Army Medical University), Chongqing, China; ^2^ Battalion 5 of Cadet Brigade, Third Military Medical University (Army Medical University), Chongqing, China

**Keywords:** GW4064, autism, sociability, BTBR, microbiota

## Abstract

Autism spectrum disorder (ASD) is considered a heterogeneous neurodevelopmental disorder characterized by significant social, communication, and behavioral impairments. The gut microbiota is increasingly considered a promising therapeutic target in ASD. Farnesoid X receptor (FXR) has recently been shown to modulate the gut microbiota. We hypothesized that FXR agonist GW4064 could ameliorate behavioral deficits in an animal model for autism: BTBR T^+^Itpr3^tf^/J (BTBR) mouse. As expected, administration of GW4064 rescued the sociability of BTBR mice in the three-chamber sociability test and male-female social reciprocal interaction test, while no effects were observed in C57BL/6J mice. We also found that GW4064 administration increased fecal microbial abundance and counteracted the common ASD phenotype of a high Firmicutes to Bacteroidetes ratio in BTBR mice. In addition, GW4064 administration reversed elevated *Lactobacillus* and decreased *Allobaculum* content in the fecal matter of BTBR animals. Our findings show that GW4064 administration alleviates social deficits in BTBR mice and modulates selective aspects of the composition of the gut microbiota, suggesting that GW4064 supplementation might prove a potential strategy for improving ASD symptoms.

## Introduction

Autism spectrum disorder (ASD) is considered a heterogeneous neurodevelopmental disorder characterized by impaired social interaction, language and communication abnormalities, and stereotypical behavior (DSM-5). The prevalence of ASD has rapidly increased in recent years, representing a significant medical and social problem (Mottron and Bzdok, 2020). There are no currently available medications to treat the core symptoms of ASD (Naviaux et al., 2017). More effective interventions are urgently needed to improve the quality of life for ASD.

Accumulating evidence shows that the gut microbiome can influence the neurobehavioral state of the host through the production of metabolites and other potential mechanisms, known as the microbiota-gut-brain axis (Clarke et al., 2013; Bruce-Keller et al., 2018). Several studies have shown altered microbiota composition and microbial metabolites in ASD patients and rodent models of ASD contribute to some of the symptoms of ASD (Needham et al., 2018; Sgritta et al., 2019; Sharon et al., 2019; Bermudez-Martin et al., 2021; Nogay and Nahikian-Nelms, 2021; Gu et al., 2022). Further, gut microbiota from ASD patients transplanted into germ-free mice is sufficient to induce hallmark behaviors of autism (Sharon et al., 2019; Xiao et al., 2021). Significantly, alteration in gut microbiota composition dramatically affects the improvement of ASD (Kang et al., 2017; Kang et al., 2019; Bermudez-Martin et al., 2021; Li et al., 2021; Nogay and Nahikian-Nelms, 2021). Several studies suggest that changes in microbiota composition induced by fecal microbiota transplantation (FMT) or probiotic treatment improve core and associated ASD symptoms (Kang et al., 2017; Goo et al., 2020; Li et al., 2021; Qi et al., 2021). Moreover, several microbial metabolites have been shown to cause social dysfunction, stereotyping, cognitive impairment, and anxiety (Vuong et al., 2017; Lobzhanidze et al., 2019; Usui et al., 2020; Mirzaei et al., 2021; Peralta-Marzal et al., 2021). Dysbiosis can cause changes in microbial metabolites that promote the core and related symptoms of ASD.

Farnesoid X receptor (FXR) is a nuclear receptor that plays a key role in fatty acid, lipoprotein, and glucose metabolism. As a receptor for bile acids (BAs), it directly or indirectly regulates intestinal microbiota composition by activating innate immunity genes in the small intestine (Ding et al., 2015; Kumari et al., 2020). The BTBR mouse, an idiopathic model for autism, recapitulates the ASD core symptoms (Careaga et al., 2015). In particular, gut microbiota alterations seemed to impair behavior (Liu et al., 2022). It has been indicated that the reduced relative abundance of certain bacterial taxa in the gut of BTBR mice was associated with the deficiency of intestinal bile acid and tryptophan metabolism (Golubeva et al., 2017). It has been confirmed that FXR agonist GW4064 prevented the development of the obese phenotype *via* inhibition of BA biosynthesis and alteration in the gut microbial composition (Zheng et al., 2017). It is supposed that GW4064 activating FXR might alleviate ASD behavioral defects *via* remodeling gut microbiota.

The present study demonstrated that the administration of FXR agonist GW4064 ameliorated the autism-associated behaviors of BTBR mice. Given the potential role of gut microbiota in autism-associated behaviors, 16S ribosomal RNA sequencing displayed GW4064 remodeled gut microbiota in BTBR mice. The present study revealed a novel role for GW4064 in controlling the balance of the microbiota community in BTBR mice. Based on the results, FXR was suggested to be a potential regulator of autism-associated behaviors and GW4064 may be used to treat ASD.

## Materials and Methods

### Animals

BTBR T+ tf/J (BTBR) mice were purchased from Jackson Laboratory (Bar Harbor, ME, USA). In order to exclude sex as an additional independent variable, only male mice were selected in this study. All mice were raised in the Third Military Medical University animal feeding facility. The mice were provided with sufficient food and water freely in a temperature-controlled light-dark cycle for 12 hours. All experiments were conducted following the approved principles of laboratory animal care and ethical approval by the Third Military Medical University. All efforts were made to reduce the number of animals used and minimize the animals’ suffering. In this experiment, we used a total of 40 mice. The document approval number is AMUWEC20223801.

### Drug Treatment

BTBR and C57BL/6J (C57), adult male mice (6-8 weeks) were intraperitoneally (i.p.) injected with GW4064 (Sigma-Aldrich) diluted to a final concentration of 30ul/g in 5% DMSO. GW4064 was administered at 30 mg/kg for 7 consecutive days. Before the start of the experiment, male mice were weaned and co-housed in cages of five each. We used a total of eight cages of mice and co-housing time was guaranteed for 4 weeks. The mice were numbered according to their weight and randomly divided into four groups: C57+DMSO, C57+ GW4064, BTBR+DMSO, and BTBR+GW4064 (n=10, for each group). Mice belonging to the same treatment group were housed together (five mice/cage). We collected mouse feces for sequencing after treatment. One day after the end of GW4064 administration, behavioral assays were performed on mice.

### Behavioral Assays

All behavioral tests were conducted during the same period of the day. Before each experiment, the mice were adapted to the experimental environment for 30 minutes, and the interval between different experiments was 24 hours.

### Three-Chambered Social Approach

The three-chambered social approach test was performed to investigate mice’s sociability, as described previously (Yang et al., 2011). Each mouse was placed separately in a rectangular socialization device (60cm x 40cm x 22cm) made of clear polycarbonate. The test consisted of two stages: the habituation and socialization phases. Test mice were placed in the central chamber during the habituation phase to freely explore the three chambers for 10 minutes. During the socialization phase, a new mouse (S) and a new object (O) were placed on each side of the chamber, and each experimental mouse was allowed to explore all three chambers for 10 minutes. The time spent in each chamber and the sniffing time (nose toward the cage at a distance of less than 2 cm) for each mouse during each 10-minute phase were detected and recorded using Noldus Observer software (Ethovision 11.0). The preference index was calculated as the difference in time between the new mouse and the new object divided by the total time spent in the two side chambers or sniffing targets (S-O/total time). The equipment was cleaned after each experiment using 70% alcohol and water.

### Male-Female Social Reciprocal Interactions

The test was performed in an apparatus (25 cm × 25 cm × 35 cm). Each experimental mouse and one estrous female mouse were left in the box for 30 minutes to acclimatize, and after 30 minutes a 5-minute recording was made using a video system. The C57 female mice were age-matched to the experimental mice with open vaginas surrounded by pink or reddish-pink tissue. The video recording system allows simultaneous recording of the social behavior of two experimental mice. We used two selected estrous females for each group tested, with each female used five times. The recorded videos were analyzed by a trained person. The time of nose-to-nose interaction between the experimental mice and females and sniffing of the body and genitalia of the females by the experimental mice (contact time longer than 2 s) were recorded using a multifunctional stopwatch. The three time durations were summed to the total socialization time. The equipment was cleaned after each experiment using 70% alcohol and water.

### Self-Grooming Test

The self-grooming test was conducted as previously described (Silverman et al., 2015). The mice were separately placed in the standard cage of the self-grooming experiment. After the mice became familiar with the test cage for 10 minutes, the observation system was applied to observe and record the time of self-grooming behavior for 10 minutes. A trained observer was blind to the drug treatment and scored the videos. The equipment was cleaned after each experiment using 70% alcohol and water.

### Marble Burying Test

A standard experimental cage (27 cm × 16.5 cm × 12.5 cm) was covered with Corncob padding about 2~3 cm thick and lightly tamped to form a flat surface with dim light conditions (~ 15 lx). Twenty black glass marbles 1.5 cm in diameter were placed on a 4 × 5 grid surface, evenly dispersed, and about 4 cm apart. Each mouse was placed in each cage for 30 minutes. We ounted the number of marbles buried > 75% after 30 minutes. The equipment was cleaned after each experiment using 70% alcohol and water.

### Open Field Test

The open-field test was performed in an apparatus (40 cm × 40 cm × 30 cm) consisting of a gray Plexiglas edge and a gray Plexiglas floor, which was used to detect general motility and anxiety in mice. The test mice were placed in the open field device from a uniform position, and the central zone time and total distance traveled for 30 minutes were recorded using the Ethovision 11.0 software. The equipment was cleaned after each experiment using 70% alcohol and water.

### Novel Object Recognition Test

We tested the short-term memory capacity of mice using the novel object recognition (NOR) test. The test was performed in an apparatus (40 cm × 40 cm × 30 cm). In the initial phase of the experiment, two identical objects (A and B) were placed on the bottom plate of the experimental apparatus, the mouse was placed into the apparatus and allowed to explore the entire arena for 10 minutes was allowed to sniff both objects, and then removed from the apparatus. After an interval of 2 hours, one of the two identical objects was replaced with the other object (B) (similar in size, but different in shape, color, and texture). The mice were placed and allowed to explore both objects freely for 10 minutes (N, time spent exploring the new object; F, time spent exploring the familiar object) and the recognition rate was calculated (N/(N+F)*100%). Noldus Observer software (Ethovision 11.0) was used to analyze. The equipment was cleaned after each experiment using 70% alcohol and water.

### Y-Maze Test

The Y-maze (three arms, 40 cm × 9 cm × 16 cm) was divided into three arms, A, B, and C, and a central area. For the test, mice were placed into the central area and allowed to explore the three arms freely for 8 minutes. The order in which the mice entered each arm was recorded. An overlapping three-arm alternation was defined as correct spontaneous alternations (SAPs). The total number of entries (N) and SAPs were monitored and calculated by the automated tracking system (Ethovision 11.0), and the alternation rate was calculated = [number of SAPs/N-2] * 100%. The equipment was cleaned after each experiment using 70% alcohol and water.

### Nest Building Task

The experiment was conducted with each mouse placed in an individually housed cage with a small number of wood shavings bedding and a square of compressed cotton nesting material close to 2.5 g/5 cm^2^ (Ancare, USA). Photos were taken at 12 hours after the experiment began, and scores based on the photo were given according to the five-point scoring criteria described previously.

### Microbiota Analysis

The 16S rRNA gene sequencing procedure was performed by Shanghai Personal Biotechnology (Shanghai, China). Briefly, total genomic DNA was extracted using the OMEGA Soil DNA Kit. DNA concentration and purity were monitored using a NanoDrop NC2000 spectrophotometer and 1% agarose gels. Amplicon generation PCR of the bacterial 16S rRNA genes V3–V4 region was performed using modified universal primer pairs. Sample-specific 7-bp barcodes were incorporated into the primers for multiplex sequencing. After PCR reaction 25 times, PCR amplicons were purified with DNA Clean Beads (Vazyme, Nanjing, China) and quantified using the Quant-iT PicoGreen dsDNA Assay Kit (Invitrogen, Carlsbad, CA, USA). After each quantification step, an equal amount of amplicon was gathered together, and 2×250 bp was sequenced using the Illumina NovaSeq platform with NovaSeq 6000 SP Reagent Kit (500 cycles). Sequences were then quality filtered, denoised, merged, and chimera removed using the DADA2 plugin. Non-singleton amplicon sequence variants (ASVs) were aligned with MAFFT and used to construct a phylogeny with fasttree2. QIIME2 and R packages (V3.2.0) were used for analysis. Alpha-diversity metrics and beta diversity metrics were estimated using the diversity plugin with samples that were rarefied to 23580 sequences per sample. Taxonomy was assigned to ASVs using the classify-sklearn naiüve Bayes taxonomy classifier in the feature-classifier plugin against the Greengenes Database. Alpha-diversity, which summarizes the diversity of microbial structure, was analyzed within a sample for several alpha-diversity metrics, including species richness (Observed), Shannon, and Chao1 using the “Phyloseq” R package. Beta-diversity, which summarizes the diversity between samples performed by the Bray-Curtis metric distance, and unweighted and weighted UniFrac distance metrics, accounts for the abundance of the operational taxonomic units. The significance of differentiation of microbiota structure among groups was assessed by permutational multivariate analysis of variance (PERMANOVA) using QIIME2. Bacterial taxonomic analyses and comparisons, including bacterial phylum and genus levels, were conducted using the Wilcoxon rank-sum test. The predominance of bacterial communities between groups was analyzed by the linear discriminant analysis (LDA) effect size (LEfSe) method. Based on the normalized relative abundance matrix, features with significantly different abundances between assigned taxa were determined by LEfSe with the Kruskal-Wallis rank-sum test (P < 0.05), and LDA was used to assess the effect size of each feature (LDA score (log10) = 2 as cut-off value).

### Data Access

All raw sequences were deposited in the NCBI Sequence Read Archive (SRA) under the accession number PRJNA821466.

Total bile acid test, real-time PCR, and prediction of microbial functions are shown in the **Supplementary Methods**.

### Statistical Analysis

All the data are presented as the mean ± standard error of the mean (SEM) and were analyzed using SPSS 25.0 software (SPSS). For the three-chambered social approach, paired t-tests were used to compare the time spent sniffing mice and objects and the time spent in the mouse-side chamber and the object-side chamber for each group of mice. For the open field experiment tests, the effects of time, mouse strain, and drug treatment were assessed using repeated-measures ANOVA (between-group factors: genotype and treatment; within-group factors: time). Two-way ANOVA with Bonferroni’s *post hoc* test was used to compare two variables. Relative abundances of specific bacteria among groups were tested using Kruskal-Wallis one-way analysis of variance by ranks. Spearman’s rank correlation coefficient was employed for the correlation analysis in the pooled datasets. For all statistical comparisons, significance was set at *P<0.05, **P<0.01, and ***P<0.001.

## Results

### GW4064 Treatment Rescued the Social Deficits in the Three-Chambered Social Approach Test Without Significant Effects on Repetitive Behaviors

**Figure 1:**
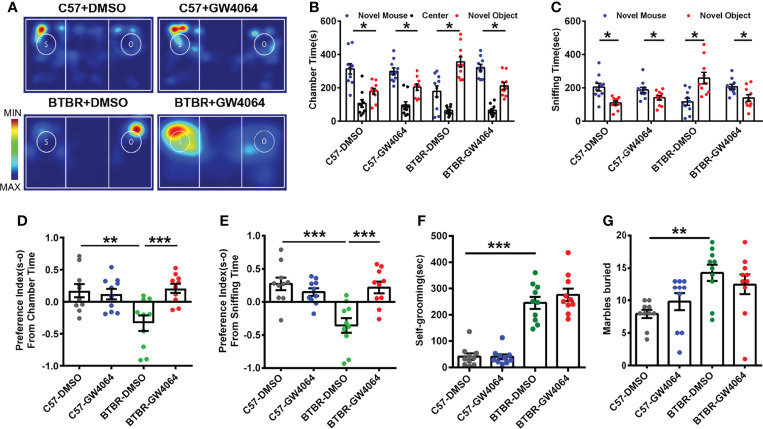
GW4064 treatment rescued the social deficits but not repetitive behaviors in BTBR mice. **(A)** Representative heat maps showing the total time and location of the BTBR and C57 mice during the 10 minute three-chambered social test. The closer the color is to red, the larger amount of time they explore the mice. “O” and “S” respectively represent the novel object and the novel mouse. **(B)** In the three-chamber test, C57 mice stayed longer in the side chamber than the novel mouse, GW4064 or DMSO administration did not affect the sociability in C57 mice. BTBR mice displayed a tendency to spend more time in the side chamber with the novel object. GW4064 treatment rescued the social deficits in BTBR mice. **(C)** In the three-chamber test, C57 mice spent more time sniffing the novel mouse. BTBR mice sniffed more on the novel object, while GW4064 treatment significantly rescued the social deficits in BTBR mice. **(D, E)** The treatment of GW4064 reversed the negative preference index from chamber time and sniffing time in BTBR mice to a similar level in C57 mice. **(F, G)** BTBR displayed a higher self-grooming time and buried more marbles than C57 mice, and the GW4064 treatment did not alter the self-grooming time and marble burying in BTBR mice. The data are presented as the mean ± SEM. n = 10. *p < 0.05, **p < 0.01, ***p < 0.001.

The three-chambered social approach test was used to determine whether GW4064 treatment effectively rescues social deficits (Amodeo et al., 2019). In the three-chambered social approach test, (**Figure 1A**) C57 mice exhibited normal sociability and spent significantly more time in the novel mouse (**Figure 1C**; paired t-test: t(9) =2.880, p=0.018) and the side chamber with the novel mouse (**Figure 1B**; paired t-test: t(9) = 2.943, p=0.016). Similar to the C57 mice, no effect of GW4064 treatment on the social ability of C57 mice in the chamber time (**Figure 1B**; paired t-test: t(9)=2.772, p=0.022) and the sniffing time (**Figure 1C**; paired t-test: t(9) = 2.351, p=0.043). Consistent with a previous study (Avraham et al., 2021), BTBR mice showed typical deficits in sociability, exhibited a reduction in chamber time for the novel mice (**Figure 1B**; paired t-test: t(9)= −2.753, p=0.022) and in sniffing time for the novel mice (**Figure 1C**; paired t-test: t(9)=−2.808, p=0.020). Nevertheless, GW4064 treatment markedly increased the exploring time (**Figure 1B**; paired t-test: t(9) =2.843, p=0.019) and sniffing time (**Figure 1C**; paired t-test: t(9) =2.270, p=0.049) of BTBR mice toward the novel mouse, suggesting the rescued impaired social ability.

To quantify alterations in sociability accurately, the social preference index for chamber time and sniffing time can be used to better assess changes in social ability. A comparison of the social preference index for chamber time using a two-way ANOVA revealed a significant difference by genotype effect (**Figure 1D**; F(1,36) = 4.693, p = 0.037) and drug effect (**Figure 1D**; F(1,36) = 6.473, p = 0.015) and genotype × drug interaction (**Figure 1D**; F(1,36) = 9.581, p = 0.004); a comparison of the social preference index for sniffing time also revealed significant differences (**Figure 1E**; genotype effect: F(1,36) =9.784, p = 0.003; drug effect: F(1,36) = 6.308, p = 0.017; genotype × drug interaction: F(1,36) = 15.218, p < 0.001). A simple effects analysis yielded that BTBR mice had significantly lower social preference indexes *via* chamber time (**Figure 1D**, p < 0.01) and sniffing time (**Figure 1E**, p < 0.001) compared to C57 mice. However, GW4064 treated BTBR mice showed significantly increased preference indexes derived from chamber time (**Figure 1D**, p < 0.001) and sniffing time (**Figure 1E**, p < 0.001) compared to DMSO treated BTBR mice. There were no significant differences in the social preference index between treatment groups in C57 mice. Of note, the mice showed no particular chamber preference during the habituation phase of the test. These tests showed that GW4064 improved the interactive social ability of BTBR mice in the three-chambered social approach test.

BTBR mice as a model of autism exhibited more obvious stereotype-like and repetitive behaviors than C57 mice (Ryu et al., 2021). We used two behaviors by detecting marble burying and self-grooming to assess stereotypic and repetitive behaviors in C57 and BTBR mice. Overall, the two-way ANOVA showed only strain differences were detected in the self-grooming test (**Figure 1F**, genotype effect: F(1,36) = 146.211, p < 0.001; drug effect: F(1,36) = 0.668, p = 0.419; genotype × drug interaction: F(1,36) = 0.751, p = 0.392) and marble-burying test (**Figure 1G**, genotype effect: F(1,36) = 13.524, p = 0.001; drug effect: F(1,36) = 0.002, p = 0.968; genotype × drug interaction: F(1,36) = 2.236, p = 0.144). In comparison with C57 mice, mice in the BTBR+DMSO group showed longer self-grooming time (**Figure 1F**, p < 0.001) and a greater number of buried marbles (**Figure 1G**, p < 0.01). Nonetheless, the amount of time self-grooming and marble burying were comparable between GW4064 treated and DMSO treated BTBR mice. As expected, GW4064 treatment had no effect on C57 mice in these tests. Behavioral data show that GW4064 treatment significantly improved the social deficits of BTBR mice but did not improve the repetitive behavior of BTBR mice.

### GW4064 Treatment Rescued the Male- Female Reciprocal Social Deficits in BTBR Mice

**Figure 2:**
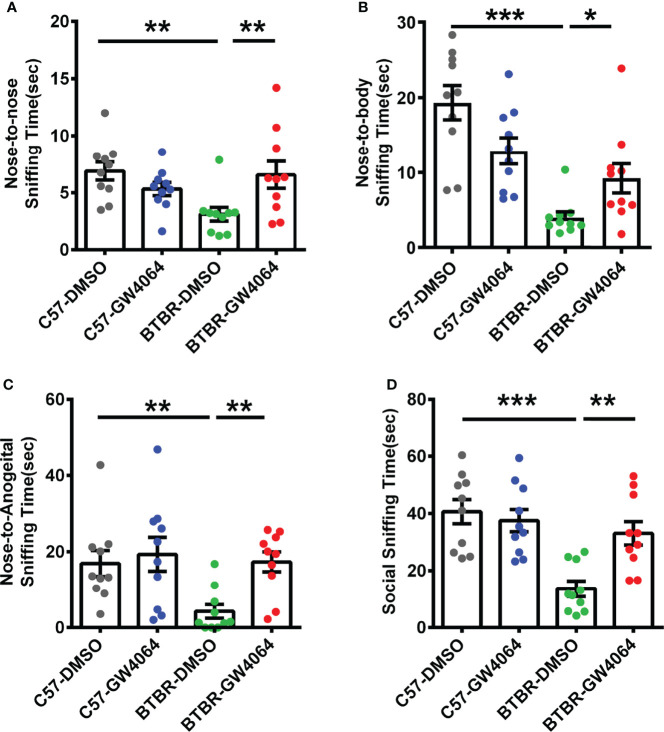
GW4064 treatment rescued the deficits in male-female reciprocal social interactions of BTBR mice. **(A–D)**. BTBR mice showed a significant lower time in the nose-to-nose **(A)**, nose-to-body **(B)**, nose-to-anogenital **(C)**, social sniffing time **(D)** than the C57 mice. The treatment of GW4064 improved the impairments in BTBR mice. The data are presented as the mean ± SEM. n= 10. *p < 0.05, **p < 0.01, ***p < 0.001.

The male-female reciprocal social test was utilized to evaluate direct sniffing behaviors in mice (Silverman et al., 2015). Two-way ANOVA revealed that there were significant effects on nose-to-nose sniffing time (**Figure 2A**; genotype effect: F(1,36) = 2.342, p = 0.135; drug effect: F(1,36) = 1.278, p = 0.166; genotype × drug interaction: F(1,36) =9.269, p = 0.004), nose-to-body (**Figure 2B**; genotype effect: F(1,36) = 28.388, p < 0.001; drug effect: F(1,36) = 0.110, p = 0.742; genotype × drug interaction: F(1,36) = 10.711, p = 0.002), nose-to-anogenital (**Figure 2C**; genotype effect: F(1,36) = 5.117, p = 0.030; drug effect: F(1,36) = 5.735, p = 0.022; genotype × drug interaction: F(1,36) = 2.718, p = 0.108), and total social sniffing time (**Figure 2D**; genotype effect: F(1,36) = 17.173, p < 0.001; drug effect: F(1,36) = 4.696, p =0.037; genotype × drug interaction: F(1,36) = 8.918, p = 0.005). *Post hoc* analysis revealed that C57 mice exhibited longer time in nose-to-nose (**Figure 2A**, p < 0.01), nose-to-body (**Figure 2B**, p < 0.001), nose-to-anogenital (**Figure 2C**, p < 0.01), and total social sniffing time (**Figure 2D**, p < 0.001) than BTBR mice. GW4064-treated BTBR mice displayed significant increases in the nose-to-nose (**Figure 2B**, p < 0.001), nose-to-body (**Figure 2B**, p < 0.05), nose-to-anogenital (**Figure 2C**, p < 0.01), and total social sniffing time (**Figure 2D**, p < 0.01). GW4064 was not detected to have any effects on C57 mice in male-female reciprocal social test.

### GW4064 Treatment Did Not Alter Performance in the Nest Building and Y-Maze Tests and Did Not Rescue Short-Term Memory Deficits in BTBR Mice

**Figure 3:**
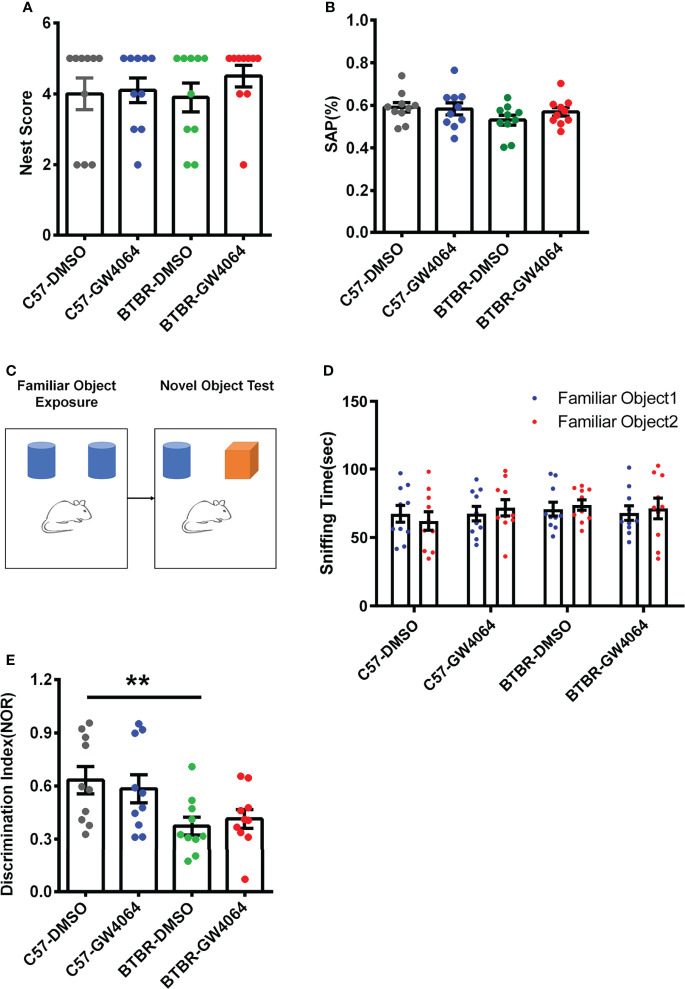
GW4064 treatment did not alter performance in the nest building and Y-maze tests and did not rescue short-term memory deficits in BTBR mice. **(A, B)** GW4064 treatment did not alter the nest scores in the nest building and the percentage of SAP in the Y-maze test. **(C)** Schematic illustration of the new object recognition task. After a period of exposure, one of the two familiar objects is replaced by another unfamiliar one, then test the time the mice stay with the novel object and the familiar object. **(D)** The sniffing time of new object recognition has no obvious preference between the familiar object 1 and the familiar object 2 during the initial learning phase. **(E)** BTBR mice displayed a lower discrimination index than C57 mice, which were not rescued by GW4064 treatment. The data are presented as the mean ± SEM. n = 10. **p < 0.01.

Nest building is a hippocampus-related congenital behavior (Zhang et al., 2018). Two-way ANOVA analyses revealed no significant effects for the genotype (**Figure 3A**; F(1,36) = 0.155, p = 0.696), drug treatment (**Figure 3A**; F(1,36) = 0.843, p = 0.365), or genotype × drug treatment (**Figure 3A**; F(1,36) = 0.430, p= 0.516) on nest scores. The Y-maze test was utilized to assess working memory (Azadi et al., 2021). Two-way ANOVA showed that no significant effects for the genotype (**Figure 3B**; F(1,36) = 2.409, p = 0.129), drug ((**Figure 3B**; F(1,36) = 0.409, p = 0.527), or genotype × drug (**Figure 3B**; F(1,36) = 0.936, p = 0.340) on SAP in the Y-maze test. These findings indicate that lack of effects of GW4064 treatment and genotype on nest scores and working memory.

The novel object recognition behavioral assay can detect and assess short-term memory capacity in mice (**Figure 3C**). During the learning phase, there was no significant difference in the exploration time of the two familiar objects, indicating that the mice had no object preference to ensure the accuracy of the testing phase (**Figure 3D**). A two-way ANOVA was used to detect differences between the four groups and revealed the presence of significant genetic effects (**Figure 3E**; F(1,36) = 10.497, p = 0.003), but no drug effects (**Figure 3E**; F(1,36) =0.003, p = 0.955) or an interaction between two factors (**Figure 3E**; F(1,36) = 0.451, p = 0.506). *Post hoc* analysis revealed significant differences in the decrease in discrimination index in BTBR mice compared to C57 mice (**Figure 3E**, p < 0.01). After GW4064 treatment, there was no significant improvement in the novel object recognition task of BTBR mice.

### GW4064 Treatment Did Not Influence Locomotion and Anxiety in BTBR Mice

**Figure 4:**
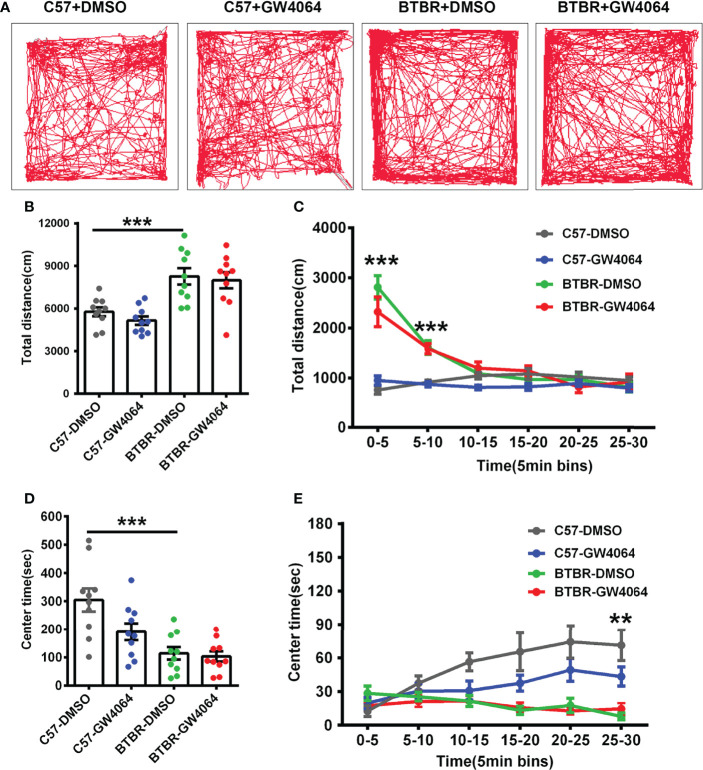
GW4064 treatment did not alter exploratory locomotion and anxiety in the open field test. **(A)** The representative traces of different groups of mice in the open field test. **(B)** The activity was measured as the total amount of distance traveled. The BTBR mice showed a stronger exploratory locomotion ability than C57 mice. **(D)** BTBR mice spent less time in the center in the open field test, and the total time of the 30-minute test in the center was not affected by GW4064 treatment in C57 and BTBR mice. **(C, E)** A 30-minute assay of distance moved **(C)** and time in the center **(E)** were computationally split into six segments with 5 minute per segment. The GW4064 treatment did not have effects on distance traveled and time spent in the center for each 5-minute segment in BTBR mice. The data are presented as the mean ± SEM. n = 10. **p < 0.01; ***p < 0.001.

Considering general locomotor activity and exploratory behavior maybe confound the detected sociability, the open-field test was performed in BTBR mice and C57 mice (**Figure 4A**). The repeated measures analysis showed that the distance traveled in each 5-minute segment decreased significantly across time (**Figure 4C**; F(5,180) = 37.605, p < 0.001), indicating the mice gradually habituated to the arena normally. The two-way ANOVA analysis revealed that there were no drug effects (**Figure 4B**; F(1,36) = 0.189, p = 0.666) or genotype × drug interactions (**Figure 4B**; F(1,36) = 0.189, p = 0.666) on the total distance traveled. However, we found a significant main effect of genotype on total travel throughout the 30-minute period (**Figure 4B**; F(1, 36) = 22.795, p < 0.001), as well as during the 0-5 minute (**Figure 4C**; F(1,36) = 73.848, p < 0.001) and 5-10 minute periods (**Figure 4C**; F(1,36) = 58.489, p < 0.001). In the open field, BTBR mice walked a longer distance in 30 minutes compared to C57 mice (**Figure 4B**, p < 0.001) as well as in the 0-5 minute (**Figure 4C**, p < 0.001) and 5-10 minute periods (**Figure 4C**, F(1,36) = 28.306, p < 0.001), suggesting that BTBR mice showed high locomotor activity within the beginning 10 minutes of the open field experiment, which is consistent with previously reported results (Reshetnikov et al., 2021).

Mice with a higher degree of anxiety preferred spending more time in the peripheral area of the open-field arena as they became adapted to the environment. The repeated measures analysis showed that the time spent in the central zone increased as the mice adapted to the environment (**Figure 4E**; F(5,180) = 3.858, p = 0.002). Two-way ANOVA showed no genotype × drug interaction (**Figure 4D**; F(1,36) = 3.098, p = 0.087) on the time spent in the center zone. However, we found drug effects (**Figure 4D**; F(1,36) = 4.554, p = 0.040) and a significant main effect of genotype and on time spent in the center zone during the 30-minute period (**Figure 4D**; F(1, 36) = 22.759, p < 0.001), as well as during the 25–30 minutes (**Figure 4E**; F(1, 44) = 28.538, p < 0.001). Compared to the C57 mice, simple effect analysis found the BTBR mice spent less time in the center zone over the total 30-minute period (**Figure 4D**, p < 0.001) as well as in the 25-30 minutes (**Figure 4E**, p < 0.01), indicating that the BTBR mice showed increased anxiety during the last 5 minutes. After GW4064 treatment, there was no significant effect on the anxiety in the open field test of BTBR mice (**Figures 4D, E**). These suggest that GW4064 treatment primarily affects socially relevant behavioral changes and plays a role independent of general changes in motor function or anxiety levels.

### GW4064 Treatment Altered the Composition of Microbiota in BTBR Mice

**Figure 5:**
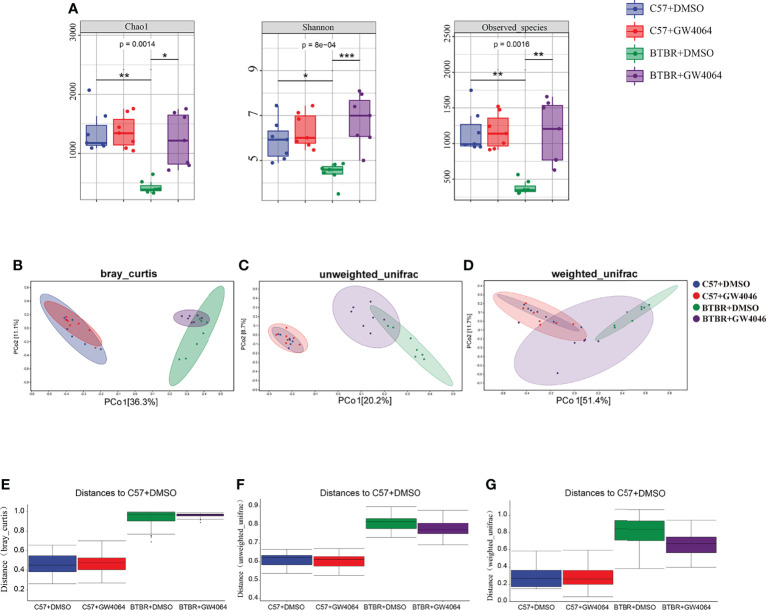
GW4064 treatment significantly modulated the decreased gut microbiota diversity in BTBR mice. **(A)** GW4064 treatment altered the alpha-diversity in BTBR mouse gut microbiota analyzed with Chao1, Shannon, Observed-species index to a similar level of C57. **(B–D)** GW4064 treatment altered the BTBR gut microbiota principal coordinate analysis (PCoA) of the beta-diversity index to get close to C57 using Bray-Curtis(B), unweighted-unifrad(C), weighted-unifrad distances(D). Each symbol represents an individual mouse. n = 7. *p < 0.05, **p < 0.01, ***p < 0.001 **(E–G)** PERMANOVA statistical tests quantify the distances among groups corresponding to the Bray-Curtis(E), unweighted-unifrad **(F)**, weighted-unifrad distances **(G)**.

Given that the disturbances to microbial populations within the gut involved abnormal behavior, the alpha and beta diversities were evaluated to determine the differences in gut microbiota diversity using 16S rRNA sequencing. The assessment of alpha diversity based on the Chao1 index, Shannon’s index, and Observed OTU indicated less microbiota diversity in BTBR mice, which is in line with the previous report on BTBR mice (Golubeva et al., 2017). GW4064 treatment led to a greater increase in alpha diversity in BTBR mice, as demonstrated by the significance seen by all three analysis methods (**Figure 5A**). We next performed principal coordinate analysis (PCoA) using the Bray-Curtis metric distance, and unweighted and weighted UniFrac distance metrics to cluster the beta diversity. There was a clear separation between C57 and BTBR groups in DMSO and GW4064 treatment (**Figures 5B–D**). The microbial composition in the mice from the BTBR+GW4064 group was more similar to that observed in mice from the BTBR+DMSO group than in those from the C57 mice. The difference analysis of the distance between the BTBR group and the C57 group showed a significant difference (**Figures 5E–G**). Weighted UniFrac-based 2D PCoA plot showed the BTBR+GW4064 group get close to the C57 group, suggesting that GW4064 treatment reduced the difference in beta diversity between the microbiota in C57 and BTBR mice (**Figures 5D, G**).

### GW4064 Treatment Altered Represented Bacterial Taxa in BTBR Mice

**Figure 6:**
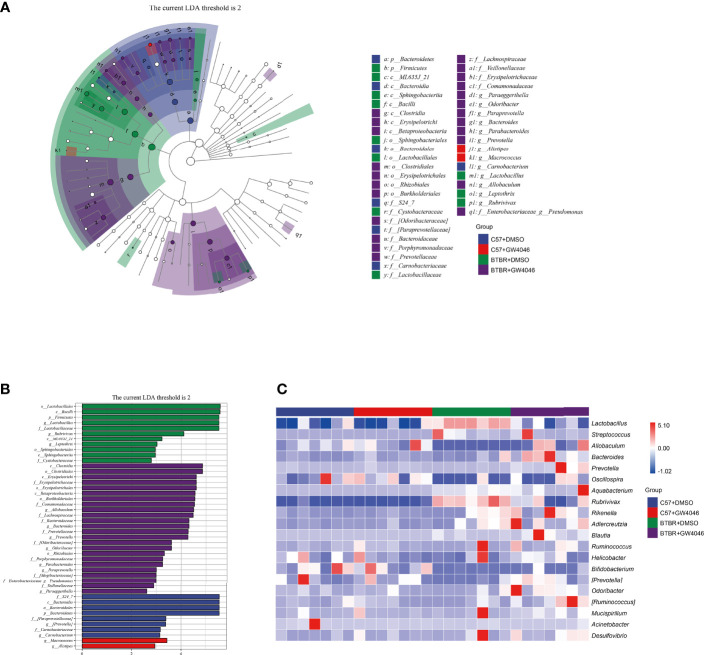
Differential taxa abundance in the gut microbiota. **(A)** LEfSe analysis of gut microbiota in mice, depicting the taxonomic association between microbiota communities among the four groups. Each node represents a specific taxonomic type. White nodes denote the taxonomic features that are not significantly differentiated. Blue, red, green, and purple nodes respectively denote the taxonomic types with more abundance than in other groups. The larger the diameter of the nodes, the more significant the difference. **(B)** The histogram of LDA value distribution and the evolutionary branch diagram of LEfSe analysis show the species whose LDA Score is higher than the set value (LDA score >2, P < 0.05). The histogram’s length represents the impact size of different species (LDA Score), and different colors represent the species in different groups (blue:C57+DMSO; red:C57+GW4064; green:BTBR+DMSO; purple:BTBR+GW4064). **(C)** Heatmap of selected most differentially abundant features at the genus level. The blue color represents less abundant, the white color represents intermediate abundance and red represents the most abundant. n = 7.

Subsequently, linear discriminant analysis effect size (LefSe) was used to identify the most differentially abundant taxa between the four groups (**Figures 6A, B**). A greater abundance in the Bacteroidetes (from the phylum to the family S24_7), was identified in C57+DMSO mice. The phylum Bacteroidetes had the LDA score of 5.549(p = 0.0001) In contrast, the relative abundance of Firmicutes (from the phylum to the genus *Lactobacillus*), Sphingobacteriia (the class and the order Sphingobacteriales), the class ML635J_21, the genus *Leptothrix*, and the genus *Rubrivivax* were higher in BTBR+DMSO group. The phylum Firmicutes had an LDA score of 5.521(p = 0.0001), as well as the genus *Lactobacillus* with an LDA score of 5.520(p = 0.0013). Nevertheless, Clostridia (from the class to the family Lachnospiraceae), Betaproteobacteria (from the class to the family Comamonadaceae), as well as Erysipelotrich*i* (from the class to the genus *Allobaculum*) displayed a relative enrichment in the BTBR+GW4064 group. The genus *Allobaculum* had an LDA score of 4.498(p = 0.0020). Based on the OTU abundance at the genus level, we also organized a comparison heatmap for the analysis of gut microbiota between the four groups (**Figure 6C**).

**Figure 7:**
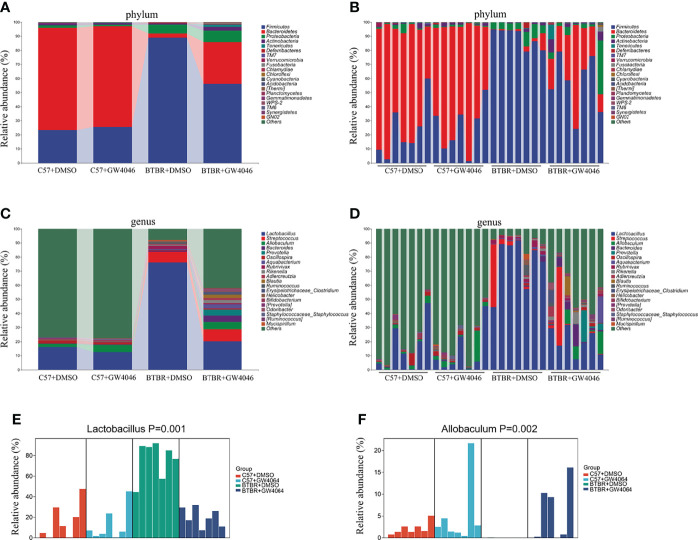
GW4064 treatment changed the autism-associated microbiota dysbiosis. **(A, B)** Bar plots of the phylum taxonomic levels among four groups (only the top 20 phylum bacteria are shown). Relative abundance is plotted for each sample (B) and the average of each group (A). **(C, D)** Bar plots of the genus taxonomic levels among four groups (only the top 20 genus bacteria are shown). Relative abundance is plotted for each sample (D) and the average of each group (C). **(E)** The relative abundance of the genus *Lactobacillus* in each sample was displayed by bar plots. **(F)** The relative abundance of genus *Allobaculum* in each sample was displayed by bar plots. n = 7.

The analysis of the gut microbiota composition at the phylum and genus levels showed specific differences between the C57 and BTBR with DMSO and GW4064 treatment. In terms of bacterial composition at the phylum level, all groups exhibited similar taxonomic communities and a relatively high abundance of the phyla Bacteroidetes and Firmicutes (**Figures 7A, B**). The relative abundance of phylum Bacteroidetes was significantly higher in C57+DMSO and C57+GW4064 groups, accounting for 78.67% and 70.29% of the gut microbiota. In the BTBR+DMSO group, the abundance of Bacteroidetes sharply declined, accounting for 7.56%. Treatment of GW4064 ameliorated the reduced Bacteroidetes in BTBR mice, accounting for 27.64%. In contrast, the relative abundance of phylum Firmicutes was significantly higher in DMSO-treated BTBR mice compared to DMSO-treated C57 mice. GW4064 treatment decreased the relative abundance of phylum Firmicutes. Taxonomic compositions at the genus level were analyzed in the four groups (**Figures 7C, D**). The relative abundance of genus *Lactobacillus* was significantly higher in the BTBR+DMSO group compared to the C57-DMSO group (**Figure 7E**, p = 0.001), and GW4064 treatment reversed this abnormal elevation (**Figure 7E**, p = 0.012). However, the relative abundance of genus *Allobaculum* was significantly lower in the DMSO-treated BTBR mice compared to DMSO-treated C57 mice (**Figure 7F**, p = 0.002), and GW4064 treatment can restore this abnormal decrease (**Figure 7F**, p = 0.005). Collectively, GW4064 treatment significantly altered the microbiota dysbiosis in BTBR mice.

### Correlation Analysis Finds Strong Associations Between Gut Microbiota and Autism-Like Behavioral Phenotypes

**Figure 8:**
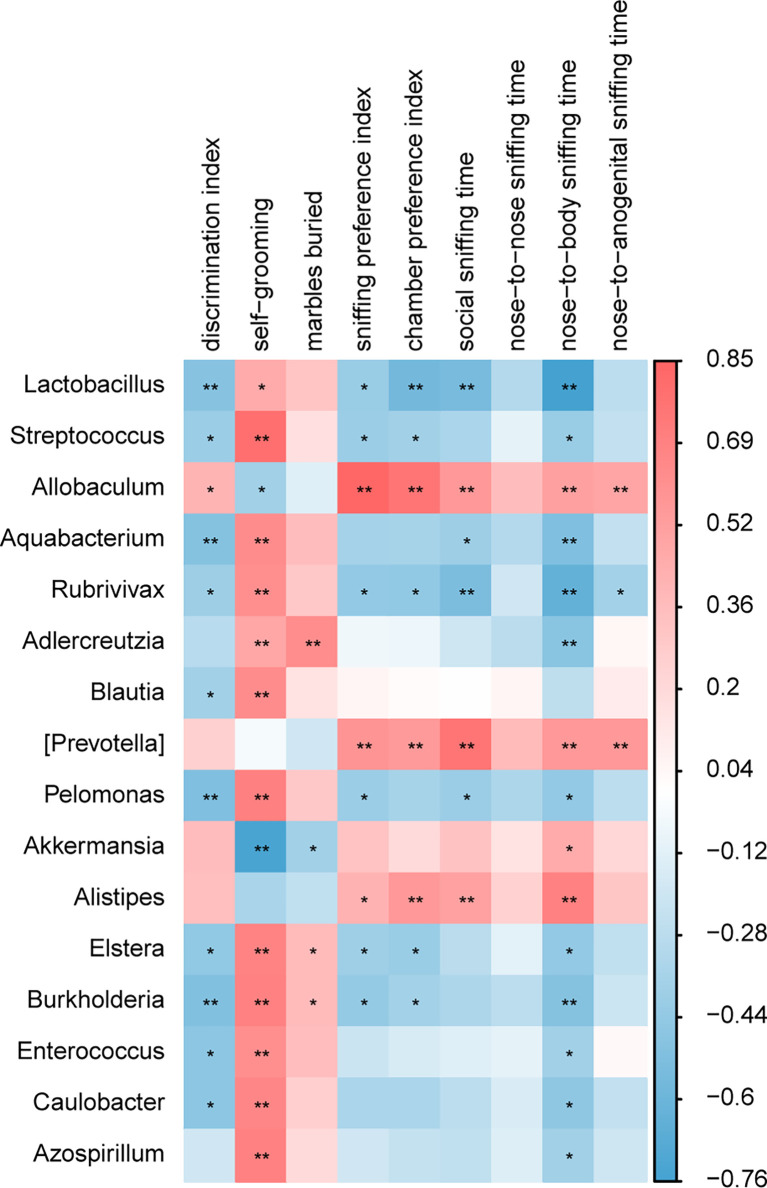
A correlation between behavioral score and gut microbiota composition. A correlation matrix showing positive (red) and negative (blue) correlations between behavioral scores and relative abundance of bacterial genera. *p < 0.05, **p < 0.01.

The correlation analysis between the relative abundance of bacterial groups and behavioral scores showed that there was a strong correlation between gut microbiota and behavioral phenotypes (**Figure 8**). A higher abundance of *Lactobacillus* in the BTBR+DMSO group negatively correlated with the social behavior scores and positively with the repetitive behaviors. *Allobaculum* was significantly lower in the DMSO treated BTBR mice and positively correlated with the social behavior scores and negatively correlated with the repetitive behaviors. Other bacteria were also associated with behavior scores, did not differ significantly between groups, and may not be the critical bacteria. We focused on the two drug-affected microbiotas (*Lactobacillus, Allobaculum*) that are involved in the behavioral changes.

### GW4064 Did Activate FXR and Altered Bile Acid Metabolism

We examined the mRNA expression of FXR in the ileum and found a significant treatment effect by a two-way ANOVA test (**Figure S2**; F(1,12) = 18.981, p = 0.001). There was no difference in FXR expression between C57 and BTBR mice, and both C57 and BTBR showed a significant rise in FXR after GW4064 treatment (p < 0.05), demonstrating that GW4064 treatment did activate FXR to function. FXR is involved in bile acid reabsorption, so we measured total bile acid levels in the plasma and the feces. Total bile acid levels in plasma were tested by two-way ANOVA and found significant differences in both genotype and drug treatment (**Figure S1** A; genotype effect: F(1,12) = 106.138, p < 0.001; drug effect: F(1,12) = 83.014, p < 0.001; genotype × drug interaction: F(1,12) =44.049, p < 0.001). Treatment with GW4064 inhibited bile acid reabsorption, with C57 mice and BTBR mice showing a significant reduction in total bile acid content in plasma after treatment. Detection of total bile acid levels in the feces revealed a significant increase in the BTBR group of mice after the treatment of GW4064 (**Figure S1** B; p < 0.001). We used PICRUST2 to enrich the biosynthetic function of different groups of gut microbiota (**Figure S3**). Among them, amino acid biosynthesis has the most obvious change. And GW4064 treatment enriched the biosynthetic function of BTBR mice more in favor of C57 mice.

## Discussion

In the present study, we demonstrated that GW4064 treatment rescued the ASD-like behaviors of BTBR mice. Specifically, we observed improvements in social behavior deficits in the three-chamber sociability test and male-female social reciprocal interaction test. Additionally, the GW4064 treatment-induced changes in behaviors were associated with the remodeling of gut microbiota in BTBR mice, evidenced by altered microbiota composition. Our study found that the changes in certain types of bacteria may be responsible for autism-associated behaviors in BTBR. Our findings reveal that GW4064 treatment may provide a novel therapeutic strategy to combat ASD core symptoms.

BTBR mice are widely used to recapitulate ASD core symptoms (McFarlane et al., 2008). As expected, the sociability of BTBR mice was impaired compared to that of C57 mice. We noticed that BTBR mice showed a significant preference for the object chamber. The disparity might be due to the injection of DSMO into BTBR mice. The disparity in the social avoidance of BTBR mice is still not well documented. Several studies showed that BTBR mice exhibited social avoidance in the chamber time test but not in the sniffing time test (Pobbe et al., 2011; Cai et al., 2017). However, some studies showed that BTBR mice never showed social avoidance (Wang et al., 2018; Cai et al., 2019a). We also noticed neonatal BTBR mice injected with DMSO as a control displayed social avoidance (Zhong et al., 2020), while adult BTBR mice injected with DMSO did not show social avoidance (Cai et al., 2019b). It seemed that social avoidance occurred in the BTBR mice in the three-chamber test is related to the age of treatment, DMSO injection, and animal numbers used in behavior tests. Importantly, GW4064 treated BTBR mice showed greater interest in the new mouse than the new object in the three-chamber social approach test. Meanwhile, GW4064 treatment alleviated the social deficits of BTBR mice in the male-female social reciprocal interaction test. Considering that social behaviors in mice could be confused by the exploratory activity and motor abilities, we further analyzed the effects of GW4064 treatment on exploratory locomotion. A standard 30-minute open-field test was applied to detect the general exploratory activity of mice. The total distance traveled by BTBR mice was not altered by GW4064 treatment, indicating that the improved social behaviors by GW4064 treatment were specific to BTBR mice. Both self-grooming and marble burying tests were measured to evaluate repetitive behavior, and we found that GW4064 treatment did not improve any of the repetitive behaviors. In addition, we also noticed that GW4064 treatment did not rescue the cognitive impairments of BTBR mice. There were no visible side effects induced by GW4064 treatment observed throughout the entire duration of the experiments. The current results revealed that the intraperitoneal administration of 30 mg/kg GW4064 for 7 consecutive days was effective in alleviating social deficits in BTBR mice.

Preclinical and clinical evidence suggests that the compositional and structural shifts of microbes and associated metabolites contribute to ASD symptoms (De Angelis et al., 2015; Mohamadkhani, 2018). Gut microbiota provides a potential therapeutic target for ASD. For instance, one study has demonstrated that *L. reuteri* rescued social deficits in ASD animal models such as BTBR mice (Sgritta et al., 2019; Kong et al., 2021). Another report found that administration of the gut microbial-derived butyrate into BTBR mice alleviated social deficits and repetitive behavior (Kratsman et al., 2016). Among the microbial metabolites linked to ASD, levels of secondary bile acids (BA) were reduced in BTBR fecal matter due to the deficits in bacteria-mediated bile conversion (Golubeva et al., 2017). Indeed, downregulation of FXR by a reduction in the luminal secondary BAs is involved in bacterial overgrowth and gut barrier dysfunction, which can in turn trigger the development of autism-associated behaviors. GW4064 treatment can comprise the deficits induced by a reduction in the luminal secondary BAs of BTBR mice, thereby remodeling gut microbiota (Golubeva et al., 2017).

Analysis of the fecal microbiota revealed a lower alpha diversity of gut microbiota in the BTBR mice, consistent with the decreased diversity in gut microbiota in autistic patients (Liu et al., 2019; Dan et al., 2020). GW4064 treatment increased the alpha-diversity of the fecal microbiota in BTBR mice, supporting a diverse gut microbiota integrity is beneficial for the host. In line with previous studies, we found marked differences in beta diversity between C57 and BTBR mice, indicating that genotype markedly transformed the biological community structures (Newell et al., 2016; Coretti et al., 2017; Golubeva et al., 2017). Alterations in β-diversity have consistently been reported in ASD murine models. Consistent with those reports, clear separation was between C57 and BTBR groups in DMSO and GW4064 treatment. Moreover, GW4064 treated BTBR mice harbored an apparent clustering separation from DMSO treated BTBR mice, indicating that GW4046 treatment also altered the biological community structures.

Several studies from ASD patients have confirmed an increase in the Firmicutes/Bacteroidetes ratio and a decrease in the abundance of Bacteroidetes in the gut microbiota (Tomova et al., 2015; Strati et al., 2017; Xu et al., 2019; Dan et al., 2020; Zafar and Habib, 2021). We noted a global alteration of fecal microbial community structure in mice from four groups. LDA and LEfSe analysis in the gut microbiota shows that Bacteroidetes were significantly enriched in C57 mice, while Firmicutes were significantly enriched in BTBR mice. Our finding is inconsistent with the study from Newell et al, showing an increase in the Bacteroidetes in fecal tissues (Newell et al., 2016). Confounding factors (treatment, sex, age) possibly lead to discrepancies in the identified changes (Rinninella et al., 2019; Manosso et al., 2021). Of note, GW4064 administration caused an increase in the relative abundance of Bacteroidetes and a decrease in the Firmicutes phylum in BTBR mice. We further highlighted selective changes in specific bacteria upon GW4064 administration. Although *Lactobacillus* bacteria are only a small member of the human colonic microbiota, the proportion of these bacteria is often positively or negatively correlated with human disease and chronic illness. Several strains in the genus *Lactobacillus* play an important role in autoimmune and other chronic diseases (Heeney et al., 2018; Fine et al., 2020). We also found a marked increase in *Lactobacillus* in BTBR mice than in C57 mice, which are considered to be probiotics. The elevation of *Lactobacillus* in the gut microflora structure has also been reported in ASD patients, as well as in ASD animal models (Adams et al., 2011; Tomova et al., 2015; Strati et al., 2017; Coretti et al., 2017; Xu et al., 2019). Correlation analysis found a negative association between increased genus *Lactobacillus* and decreased social deficits in BTBR mice. GW4046 treatment improved social deficits might be related to the reduced relative abundance of *Lactobacillus* in BTBR mice. Nonetheless, studies also found supplementation with *Lactobacillus reuteri* could alleviate ASD-like behaviors (Sgritta et al., 2019). Given the conflicting reports, it seemed that the increase of *Lactobacillus* may contain a complex relationship of bacterial flora structure, which needs to be further investigated.

Interestingly, our study revealed a significant reduction of genus *Allobaculum* in the microbiotas of BTBR mice, which could be increased by GW4046 treatment. *Allobaculum* is a mouse commensal and associated with 5-hydroxytryptamine levels and short-chain fatty acid production (Wu et al., 2020; Wang et al., 2020; Li et al., 2020). Several lines of studies have found that the reduction of genus *Allobaculum* is involved in behavior deficits and neurological disease (Davis et al., 2017; Gubert et al., 2020; Xie et al., 2020; Gallucci et al., 2021). Correlation analysis found a positive association between decreased genus *Allobaculum* and decreased social deficits in BTBR mice. It is supposed that increased *Allobaculum* induced by GW4046 treatment drives social deficit improvement.

However, we cannot provide clear evidence that GW4064 treatment contributes to the rescued social deficits of BTBR mice *via* gut-brain signaling due to a lack of pretreatment behavioral and 16s analysis. In addition, a limitation of the study is that they were conducted exclusively on male mice. Female mice are included in a future study to determine if microbiota and behavioral alterations respond to GW4064 treatment in a sex-dependent manner.

## Conclusions

These data demonstrate that GW4046 treatment alleviates social deficits and remodels gut microbiota in BTBR mice. These results highlight the notion of GW4046 as a potential intervention to positively modulate the microbiota-gut-brain axis and show that GW4046 supplementation might prove a viable strategy in improving specific core symptoms in ASD. Moreover, it further confirms that the potential of microbiota remodeling is involved in mental health recovery.

## Data Availability Statement

The datasets presented in this study can be found in online repositories. The names of the repository/repositories and accession number(s) can be found below: https://www.ncbi.nlm.nih.gov/bioproject/PRJNA821466, PRJNA821466.

## Ethics Statement

The animal study was reviewed and approved by Laboratory Animal Welfare and Ethics Committee of Third Military Medical University.

## Author Contributions

XF, TL, and JL designed the study; TL and JL analyzed the data; XF, TL, and JL wrote the manuscript; and TL, JL, CL, ZG, LZ, JG, and YL performed the experiments. XF and TL provided technical and financial support. All authors read and approved the final manuscript.

## Funding

This work was supported by the National Nature Science Foundation of China (No. 82071544), the Natural Science Foundation Project of Chongqing (NO. cstc2020 jcyj-msxmX0816), and the Student’s Platform for Innovation and Entrepreneurship Training Program (202190035017).

## Conflict of Interest

The authors declare that the research was conducted in the absence of any commercial or financial relationships that could be construed as a potential conflict of interest.

## Publisher’s Note

All claims expressed in this article are solely those of the authors and do not necessarily represent those of their affiliated organizations, or those of the publisher, the editors and the reviewers. Any product that may be evaluated in this article, or claim that may be made by its manufacturer, is not guaranteed or endorsed by the publisher.
