# Supplementary material for: GW4064 Alters Gut Microbiota Composition and Counteracts Autism-Associated Behaviors in BTBR T+tf/J Mice
Source: Front Cell Infect Microbiol. 2022 Jun 22;12:911259. doi: 10.3389/fcimb.2022.911259 (PMC9257030; doi:10.3389/fcimb.2022.911259)
Supplement: Supplementary file 1 [file DataSheet_1.docx]

Supplementary Materials and Methods

# Supplementary Data

**MATERIALS AND METHODS**

**Total Bile Acid Test**

Blood samples from mice were collected and centrifuged (3000 rpm; 15 min; 4 °C), and the supernatant plasma was collected for the assay. Mouse fecal samples were collected about 1g, homogenized by adding 9ml of PBS (phosphate buffered solution), centrifuged (5000 rpm; 15 min; 4 °C) and the supernatant was taken for the assay. Samples were assayed using the Total Bile Acid (TBA) Colorimetric Assay Kit (Jiangsu Meibiao Biotechnology, China). All steps followed the manufacturer’s instructions.

**Real-time PCR**

Total RNA from ileal tissue was extracted using the Ultrapure RNA kit (CWBIO) and reverse transcribed to cDNA at competent concentrations according to the instructions of the PrimeScript RT kit (Takara). qRT-PCR was performed and analyzed by the CFX96 Real-Time PCR system (Bio-Rad). The average relative levels of target mRNAs were normalized to GAPDH expression levels. The primer sequences used are as follows: mouse FXR—forward primer: 5′-GGCAGAATCTGGATTTGGAATCG-3′ and reverse primer: 5′-GCCCAGGTTGGAATAGTAAGACG-3′; and mouse GAPDH—forward primer: 5′-AGGTCGGTGTGAACGGATTTG-3′ and reverse primer: 5′-TGTAGACCATGTAGTTGAGGTCA-3′.

**Prediction of Microbial functions**

Microbial functions were predicted by PICRUSt2 (Phylogenetic investigation of communities by reconstruction of unobserved states) upon MetaCyc (https://metacyc.org/)databases.

# Supplementary Figures


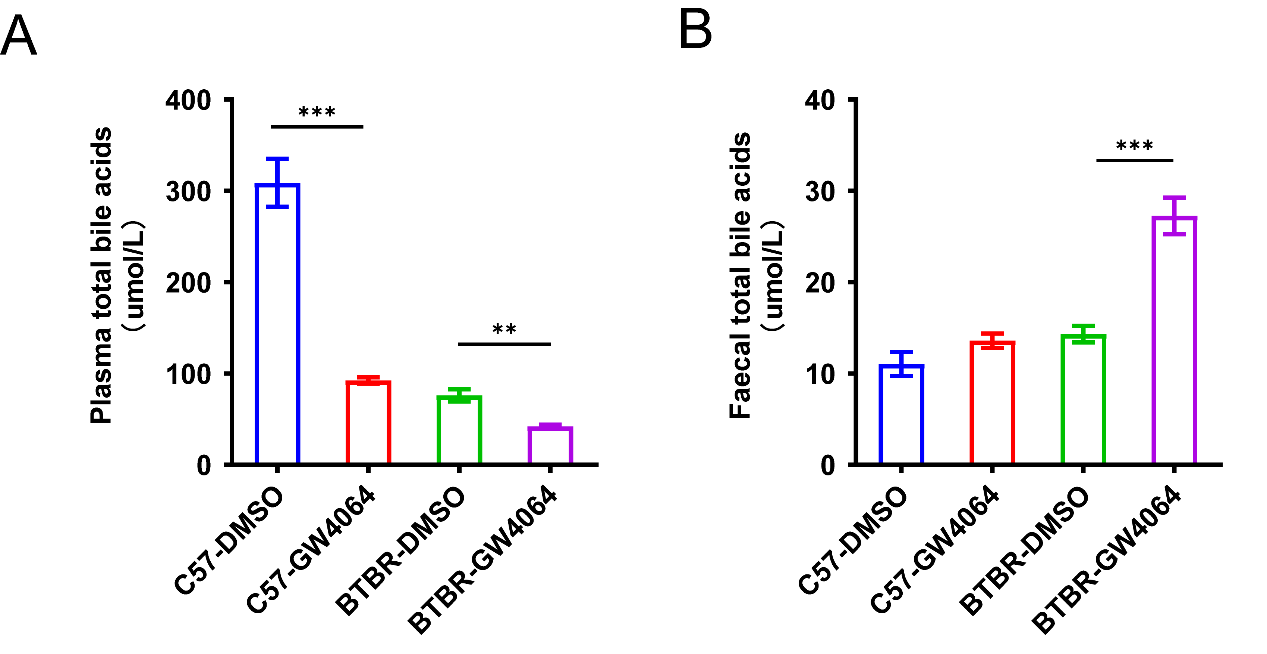


**Supplementary Figure 1. GW4064 can change the concentration of total bile acid in plasma and feces.** (A) Plasma total bile acid content decreased in BTBR mice, and GW4064 treatment can reduce plasma total bile acid concentration. (B) GW4064 significantly increased the content of total bile acid in BTBR mice after treatment. n = 4. *p < 0.05, **p < 0.01, ***p<0.001.

**
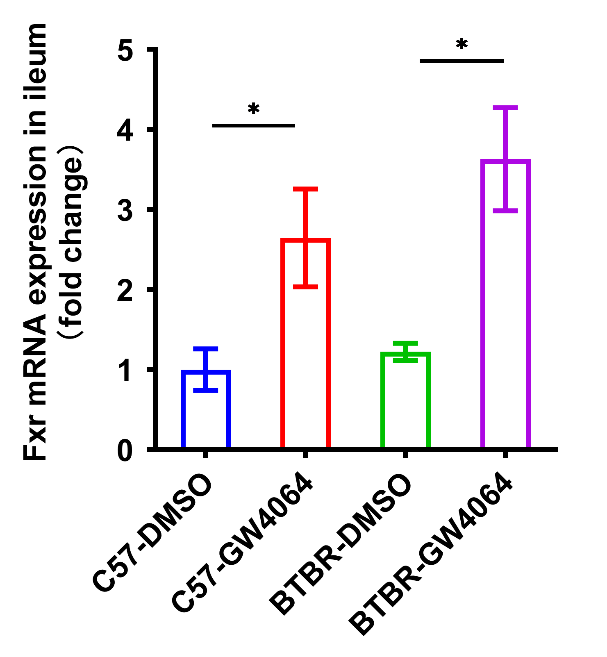
**

**Supplementary Figure 2.** **FXR gene** **expression in ileum tissue.** The FXR agonist GW4064 increased the mRNA expression of the FXR gene. n = 4. *p < 0.05, **p < 0.01, ***p<0.001.


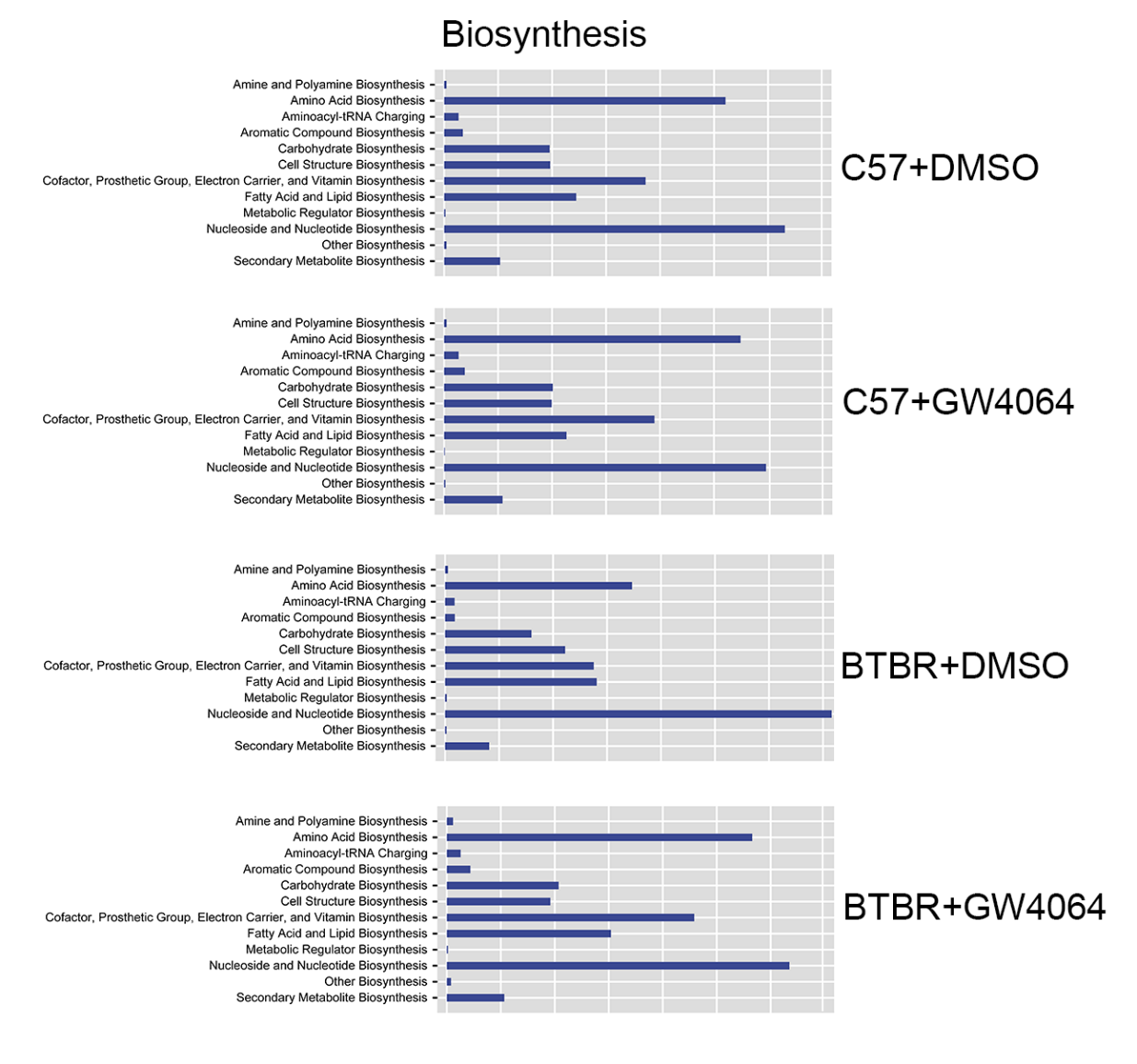


**Supplementary Figure 3. PICRUST2 was used to enrich the microbiota of different groups of mice under biosynthetic items.** GW4064 treatment enriched the biosynthetic function of BTBR mice more in favor of C57 mice.
